# 1657. Evaluating the Effectiveness of Biofire® FilmArray® BCID2 Sepsis Panel in Pediatric Antibiotic Stewardship Program: Less time and more control

**DOI:** 10.1093/ofid/ofad500.1490

**Published:** 2023-11-27

**Authors:** Juan Pablo Londono-Ruiz, Juan Pablo Betancur, Stephanie Casadiego-Payares, Daniela Castellanos-Leguizamon, Ivan Felipe Gutierrez-Tobar

**Affiliations:** Clinica Infantil Colsubsidio, Staphylored Colombia, Bogota, Distrito Capital de Bogota, Colombia; Universidad CES, Medellin, Antioquia, Colombia; Universidad CES, Medellin, Antioquia, Colombia; Universidad de los Andes, Bogota, Distrito Capital de Bogota, Colombia; 4Clínica Infantil Santa Maria del Lago y Clinica Infantil Colsubsidio, Bogota, Distrito Capital de Bogota, Colombia

## Abstract

**Background:**

The use of molecular biology tools has allowed for a decrease in the time required for effective therapy in bacteremia. Integrating these tools into Antimicrobial Stewardship Programs (ASP) can improve clinical outcomes. The aim of this study is to describe the impact of using a molecular biology platform for sepsis as part of an ASP in a pediatric hospital.

**Methods:**

This descriptive study collected data from positive blood cultures that underwent Biofire® FilmArray® BCID2 testing for sepsis from January 2021 to February 2023. In our hospital, taking this molecular test is indicated in all blood cultures positive for Gram-negative bacilli, in Gram-positive cocci only when there is a pyogenic focus or both sets are positives, additionally in patients with immunosuppression or who present with septic shock. Clinical and microbiological characteristics of the samples were described, along with the impact of the ASP after receiving the test results. Data analysis was performed using the R program.

**Results:**

A total of 119 positive blood culture bottles were included in the study, in which the molecular test was performed. Gram-positive cocci were identified in 82 (68.9%) bottles, while Gram-negative bacilli were identified in 37 (31.1%). The mean detection time for Gram-positive cocci was 13 hours and 33 minutes (range: 11-18 h), while for Gram-negative bacilli it was 11 hours and 30 minutes (range: 9-13 h). The most frequently detected germs are presented in Table 1. Among the 119 patients, the FilmArray test result prompted a change in antibiotic therapy in 54 cases (45.3%). In an additional 28 cases (23%), a change was made after the final culture result. Only 6 patients (5%) experienced clinical deterioration after the FilmArray® test, requiring changes prior to the definitive culture result.

**Table 1:**
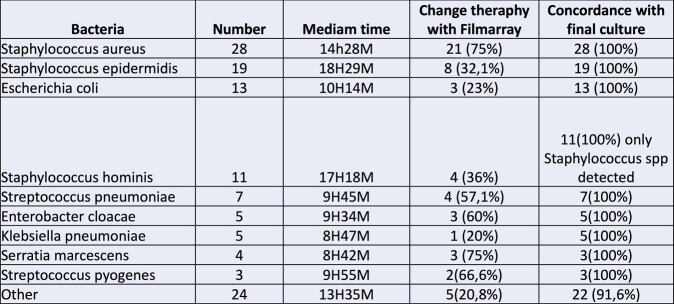
Different microorganisms, diagnosis times and concordance with the definitive culture

**Conclusion:**

In conclusion, our study demonstrates that the use of molecular tools in positive blood culture bottles, guided by an ASP, allowed for rapid changes in nearly half of the cases. Few patients experienced deterioration after the completion of the FilmArray® test, requiring empirical changes in the antibiotic. These findings suggest that incorporating molecular tests into ASPs can significantly improve the management of bacteremia, leading to more effective and timely treatment decisions.

**Disclosures:**

**All Authors**: No reported disclosures

